# Ant Guild Identity Determines Seed Fate at the Post-Removal Seed Dispersal Stages of a Desert Perennial

**DOI:** 10.3390/insects12020147

**Published:** 2021-02-08

**Authors:** Gilad Ben-Zvi, Merav Seifan, Itamar Giladi

**Affiliations:** 1 Sede Boqer Campus, Albert Katz International School for Desert Studies, Jacob Blaustein Institutes for Desert Research, Ben Gurion University of the Negev, Midreshet Ben-Gurion 8499000, Israel; giladbenzvi@hotmail.com; 2 Sede Boqer Campus, Mitrani Department of Desert Ecology, Swiss Institute for Dryland Environmental and Energy Research, The Jacob Blaustein Institutes for Desert Research, Ben-Gurion University of the Negev, Midreshet Ben-Gurion 8499000, Israel; seifan@bgu.ac.il

**Keywords:** granivorous ants, scavenger ants, elaiosomes, seed redispersal, *Sternbergia clusiana*, *Messor* sp., *Cataglyphis* sp., directed dispersal

## Abstract

**Simple Summary:**

Ants play a dual role in their interaction with plant seeds. Many ant species, mainly harvester ants, consume plant seeds (granivory), whereas other ants, mainly scavengers, provide a beneficial seed dispersal service. Granivory by ants is frequently documented in deserts, but beneficial seed dispersal is rarely studied in such ecosystems. We followed the handling of seeds of *Sternbergia clusiana*, an ant-dispersed plant, by two guilds of desert ants. We focused on the treatment of seeds within the nest and on the redispersal of seeds after ejection from the nest. Scavenging ants rarely consumed the seed itself, they deposited most seeds away from the nest in apparently suitable microhabitats. In addition, we found that most of the seeds that were relocated by scavenging ants arrived at sites under the canopy of shrubs. Such sites might be beneficial for the establishment and success of plants in the arid environment. Indeed, we found that the subject plant was more likely to be found under shrubs than elsewhere, which suggests that it benefits from being placed there. Such documentation of seed dispersal activity by scavenging ants in arid ecosystems suggests they may be efficient seed dispersers in deserts, as they are in other ecosystems.

**Abstract:**

Ants play a dual role in their interaction with plant seeds. In deserts, the consumption of seeds by granivorous ants is common, whereas mutualistic seed dispersal, often associated with scavenging ants, is rarely documented. We evaluated the contribution of both ant guilds to efficient seed dispersal of an ant-dispersed plant, *Sternbergia clusiana*, in a desert ecosystem. We presented seed to colonies of three species of desert ants from the *Cataglyphis* (scavengers) and *Messor* (granivorous) genera. We recorded seed consumption, ejection from the nest, and seed transportation to potentially beneficial microhabitats. We evaluated microhabitat quality by testing the association between habitat types and the plant at various life stages. As expected, granivores mainly consumed the seeds, whereas scavengers consumed the elaiosome (seed appendage serving as a reward), but left the seeds intact. Moreover, scavenging ants relocated the seeds much further than granivores, mainly to shrub patches. The disproportional distribution of the plant under shrubs at several life stages suggests that this microhabitat is beneficial for the plant. Overall, while granivores seem to mainly harm seed dispersal, we provide the first evidence for the beneficial contribution of scavenging ants in deserts, showing they exhibit the same suite of characteristics that render them efficient seed dispersers in other ecosystems.

## 1. Introduction

Ants are an extremely common and diverse group of arthropods that have immense effects on ecosystem processes [1]. Ants are considered a major ecosystem engineer via the construction of their nests and the associated resource re-distribution, an effect that is apparent in arid ecosystems [2,3]. In addition, ants exert a more direct effect on plant communities via seed removal and seed dispersal [4,5]. While the interaction of ants with seeds generally varies from pure granivory to mutualism [6], in desert ecosystems the most commonly studied ant–seed interaction is granivory [7,8]. The potential role of ants as beneficial seed dispersers in deserts has hardly received any attention.

Myrmecochory is a term used to describe a mutualistic interaction of seed dispersal by ants that is characterized by the production of an elaiosome, a lipid-rich seed appendage that attracts and elicits seed carrying behavior in certain groups of ants [9,10]. Treated for years as a diffuse mutualism, myrmecochory has been recently recognized as an asymmetric, functionally specific and context- and partner-dependent interaction [11,12,13]. Myrmecochory, in the narrow sense of elaiosome-mediated seed dispersal, has been thoroughly studied in various ecosystems, but rarely in deserts [13,14,15].

Two ant guilds may interact with seeds in desert environments. Granivorous ants consume the seeds of many plants species [5,16,17] and are thus considered seed predators. However, infrequently, seeds that are carried by granivorous ants may escape predation and be effectively dispersed, either along foraging trails or at refuse piles [6,18,19]. Harvester ants, which are among the more dominant members of the granivore guild, tend to exhibit little or no affinity to the presence or size of an elaiosome on a seed [20,21], thus dispersing myrmecochorous seeds only occasionally. Scavenging ants, on the other hand, ignore the seeds of most plant species, but exhibit high affinity for myrmecochorous seeds, which they transport over long-distances, bring to their nest, and leave intact after removing the elaiosome [13]. Generally, scavenging ants are considered effective seed dispersers that positively affect plant fitness [22,23], though little is known about their interaction with seeds in arid ecosystems.

The effect of ants, as seed dispersers, on the plant success may be manifested at various phases of the seed dispersal process (from seed removal until germination). Moreover, the long-term outcome of dispersal events may vary according to the conditions that a germinating plant experiences later in its life [24]. Three main phases of the seed dispersal process may affect seed fate. The first phase involves seed removal and transport towards and into the ant nest. The second involves the handling of seeds within the nest. While in the nest, the seed may be consumed, thus losing its recruitment potential, or only the elaiosome is removed while the seed is left intact, a process that potentially reduces further predation risk and benefits germination success [25,26,27]. Seeds that were not consumed may be stored in the nest or ejected. The handling of seeds in the nest is sometimes followed by a third phase, redispersal, that involves the transport of ejected seeds to considerable distances away from the nest entrance [28,29]. While seed removal and transport (the first phase) has been extensively studied, the two post-removal dispersal stages, handling of seeds within the nest and redispersal, have rarely been documented [30,31]. Moreover, we know almost nothing about the effect of ant guild on the consequences of these two neglected phases of the dispersal process.

The benefits that plants receive from being dispersed by ants may be modified at each of the phases and vary with the behavior of the ant species. The benefits to the plants are classified by several non-mutually exclusive categories [9]. These include the reduction of intraspecific and kin competition, the reduction of seed predation (predator avoidance), and/or the non-random dispersal of seeds to microsites of high establishment probability (i.e., directed dispersal) [22,23,32]. With respect to the directed dispersal hypothesis, benefits are usually evaluated while assuming that the final destination of seed is the vicinity of the nest, specifically the nest midden [22,32,33]. Consequently, the benefits to the plant that are associated with this hypothesis are linked to the midden environmental conditions. However, whenever seeds are being redispersed from the midden, the costs and benefits of directed dispersal should be evaluated at the final seed deposition site, which can no longer be assumed to be the midden [23,28,29,31]. The environmental conditions and seed aggregation levels at the deposition site will depend, among other things, on whether redispersed seeds are scattered randomly by the redipsersing ant or are disproportionally relocated to specific microsites [28,29].

The aim of this study was to compare the contribution of two common desert ant guilds to elaiosome-mediated seed dispersal process of *Sternbergia clusiana* (Amaryllidaceae), a perennial myrmecochore that reaches its southern distribution edge in the Negev desert in Israel. We focused on the two post-removal phases: (1) within-nest handling of seeds, specifically, seed predation and elaiosome removal, and (2) the extent of redispersal by the two ant guilds and an evaluation of whether redispersal is random, non-random or even directed. The seeds of *S. clusiana* have been shown to be removed by both granivorous and scavenging ants, though only scavenging ants show clear affinity and preference for these seeds over seeds of other plants [20,34]. Preliminary observations suggested that redispersal by scavenging ants is non-random and potentially directed to microsites under shrubs. As shrub understory in many arid environment serves as a “fertile island” and enhances the establishment of (mainly) herbaceous plants [35,36], we hypothesize that it may also benefit the establishment of *S. clusiana*. If that is indeed the case, then a non-random redispersal of its seeds towards shrubs by the scavenging ants may be the first example of redispersal of seeds by ants that is also directed to preferred sites.

## 2. Materials and Methods

### 2.1. Study Species

We followed the seed dispersal and seed handling behavior of three ant species that are common in the Negev desert and are candidate dispersers of *S. clusiana*. These ant species included two granivore species (*Messor ebeninus* and *Messor arenarius*), representing different foraging strategies and levels of within-species variation in worker size, and a large scavenging species, *Cataglyphis savignyi* [37,38]. *Messor ebeninus* forages mainly along trunk trails with up to a few thousand workers at a time, and worker size varies from 3.5–13 mm body length. The nest has no discernible mound, but has a distinctive midden with an outer circle of seed particles and other types of waste and debris that can reach a 3-m diameter [38,39]. The other granivore, *M. arenarius*, mainly forages solitarily, but occasionally exhibits modest recruitment along pheromone trails, with up to 100 workers at a time. The worker size ranges from 4–18 mm, but almost all foraging individuals are 13–18 mm long [38,39,40]. The nest has at least one mound (at other seasons it sometimes has two openings with two mounds several meters apart), which is 0.4–0.8 m wide [38,39] and contains seed particles and other waste and debris. *Cataglyphis savignyi* is a large scavenging ant with worker size at a range of 5–13 mm, and the foraging strategy is strictly solitary. It rarely collects or carries any plant materials, seeds with elaiosome being an exception. The nest’s mound, which is seasonal, does not serve as a midden and is the smallest in diameter among the three species—up to 0.4 m [38].

*Sternbergia clusiana* is a bulbous herbaceous perennial myrmecochore that flowers in autumn, sets leaves in winter, and develops fruits and seeds that mature in spring [41,42]. Each of its seeds is equipped with a very large elaiosome. The diaspore (seed + elaiosome) mass (109.6 ± 40.5 mg, $\bar{X}\pm SD$), as well as the elaiosome/seed mass ratio (0.99 ± 0.24, $\bar{X}\pm SD$), are some of the highest among the myrmecochore species of the Mediterranean region, indicating a high investment in an attractive reward. This investment has been shown to mainly attract large scavenging ants of the *Cataglyphis* genus, but granivore species of the *Messor* genus have also been observed interacting with *S. clusiana*’s seeds [20,34].

### 2.2. Study Site and Redispersal Experiment

To assess post-removal behavior by each of the ant species, a seed manipulation and redispersal experiment was conducted in spring 2017 on the Tzin Plateau in the Negev Desert, Israel (34°46′41′′ E 30°51′26′′ N). The site is an arid plateau dominated by short annual plants with sparse perennial vegetation (shrub cover <10%; see [43]), making seed detection near and away from the nest relatively easy. Thirty nests, ten of each ant species, were located and marked. At the onset of the experiment, a seed depot of 10 tagged seeds was placed near the entrance of each nest, and was observed until all seeds were taken into the nest. Following Canner and Spence [44], the seeds were tagged using 0.5 mm long coded wire tags (CWTs, Northwest Marine Technologies, Inc., Shaw Island, WA), which were attached to the hard seed coat with UHU superglue (UHU GmbH & Co. KG, Bühl/Baden, Germany). Preliminary cafeteria experiments, where seeds are presented to foraging ants and their seed-tending behavior is recorded, showed that (a) the presence of a tag on a seed did not affect seed collection or removal by any of the ant species; (b) the ejection of seeds from the nest usually started within a day, but could last for up to two weeks; and (c) at the end of this two-week period, the majority (>70%) of the seeds that were taken into a nest were detected, either next to the nest opening or after being redispersed. Thus, after allowing at least 24 h for each ant colony to process the seeds within the nest, a Handheld T-Wand Metal Detector (Northwest Marine Technologies, Inc., Anacortes, Washington State, USA) was used to search for tagged seeds in an area of 40 × 40 m with the nest at its center. This detection protocol, which lasted for 1 h around each nest, was repeated 1, 2, 3, 4, 7, and 14 days after the presentation of seeds to the ants. A tagged seed could be detected even if it was buried in the soil, as long as the detector was within 5.5 cm of the tag. For each detected seed we documented (a) whether the seed was partially consumed and/or whether the elaiosome has been removed, (b) the distance and direction from the nest entrance, (c) whether the seed was deposited locally (<2 m from the nest entrance–mean radius of *Messor’s* midden) or was redispersed (>2 m from the nest entrance, redispersed), and (d) whether a seed was placed under a shrub or in the open. This latter distinction is based on an early realization that *Cataglyphis* ants use shrub patches as preferred seed deposition sites. At the end of the two weeks detection period, we tallied, separately for each nest, the number of seeds that were in the categories listed above.

### 2.3. The Distribution of Sternbergia clusiana with Respect to the Distribution of Shrubs

Dispersal is defined as directed when seeds arrive disproportionally to specific microhabitats, and when these microhabitats have positive effects on survival and establishment [32]. Testing whether any of the ant species disperse seeds disproportionally to a specific habitat type (shrub patch) is relatively easy and was included as part of the experiment described above. Ideally, testing whether such habitat also confers long-term benefits to the plant should include following the plant performance through its whole life span. This is an extremely difficult task in the case of a plant species with such a long and slow life cycle, such as *S. clusiana*. Rather, we took advantage of a large-scale census of *S. clusiana*, where local distribution in relation to shrub cover has been recorded. The data originated from three desert sites (Table A1), where natural populations of *S. clusiana* were monitored. In each of these sites, a grid of 60–100 1 × 1 m plots was established and overlaid to roughly cover the extent of a local population of *S. clusiana*. Thus, the exact number of 1 × 1 m plots varied slightly among sites. In each of these plots, we recorded the proportion of area covered by shrub, as well as the number of seedlings, mature and mature fruiting plants, representing germination, establishment, and reproduction, respectively. For each individual, we noted whether it was under a shrub or in an open habitat. Based on these data, for each plot and for each life stage, we calculated the number of individuals that are expected to be under a shrub if the plant distribution is independent of a shrub’s presence. This was estimated by multiplying the number of individuals of each life stage in a plot by the proportion of shrub cover measured in that plot.

### 2.4. Statistical Analysis

We used a series of GLMs to test the effects of ant species, as a fixed explanatory variable, on various phases and aspects of seed dispersal. We used a binomial distribution and a logit link function for the analysis of seed predation, elaiosome removal, redispersal (the arrival of the seeds to the midden or beyond), and habitat type to which a seed arrives (underneath a shrub or open habitat). We used a normal distribution and a log link function for the analyses of redispersal distances.

For each life stage, we tested, using a paired-sampled *t*-test, the within plot observed vs. expected number of individuals under shrub. For each analysis, we considered only those plots that had at least one *S. clusiana* individual of the respective life stage; thus, there are different degrees of freedom for the various life stages. All analyses were executed in SPSS 25 [45]. All graphics were executed in R software 3.4.3 [46] using R packages lattice [47] and plotrix [48].

## 3. Results

### 3.1. Redispersal Experiment

**Figure 1:**
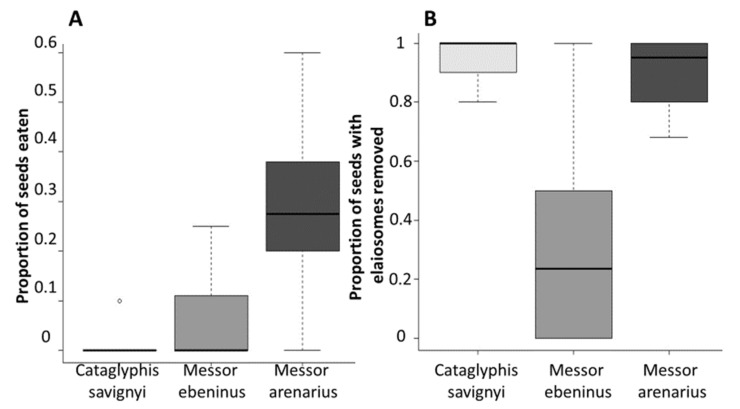
Seed condition following within-nest treatment by each ant species: (**A**) seed predation across species. (**B**) Elaiosome removal across species. Each boxplot represents the median (black horizontal line), 25, and 75 quartiles. The error lines represent the maximal and minimal values, unless there are suspected outliers (unfilled circles): in this case, they represent the “inner fence” (1.5 × likely range of variation from the quartile).

Most (83.67%) of the tagged seeds were recovered during the two weeks that followed their initial presentation to the ants. The percentage of recovered seeds was 94%, 78% and 79% for *C. savignyi*, *M. ebeninus,* and *M. arenarius*, respectively. The recovered seeds included intact seeds with the elaiosome still attached (i.e., the whole diaspore), intact seeds where only the elaiosome had been removed, and seeds that were partially consumed. Seed condition following their handling within the nest differed among ant species. *Messor arenarius* was the only significant seed predator, consuming 31.65% of the seeds that were retrieved, significantly more than the two other ant species ($\chi_{\left(2\right)}^{2}\;=\;25.07,\;p<0.001$, Figure 1, Table A2 and Table A3). Elaiosome removal also varied significantly across the ant species ($\chi_{\left(2\right)}^{2}\;=\;85.74,\;p<0.001$, Figure 1, Table A2 and Table A3), with 94.68%, 23.08%, and 91.13% of the retrieved seeds having their elaiosome removed by *C. savignyi*, *M. ebeninus*, and *M. arenarius*, respectively.

**Figure 2:**
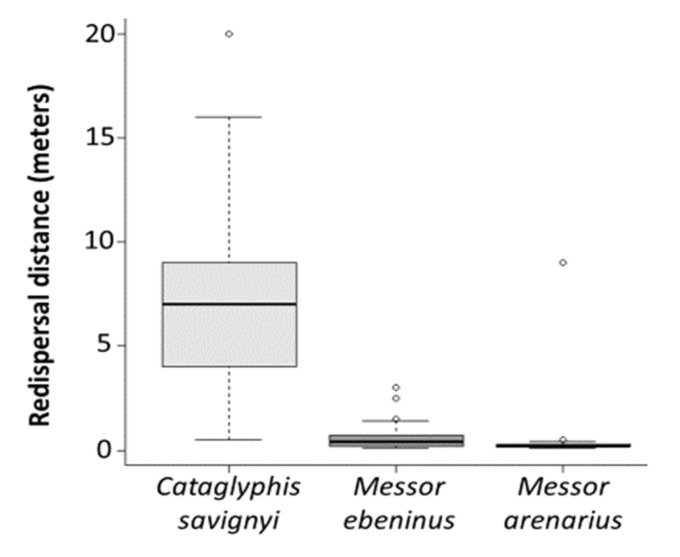
Seed redispersal distance by each ant species. Each boxplot represents the median (black horizontal line), 25, and 75 quartiles. The error lines as described in Figure 1.

**Figure 3:**
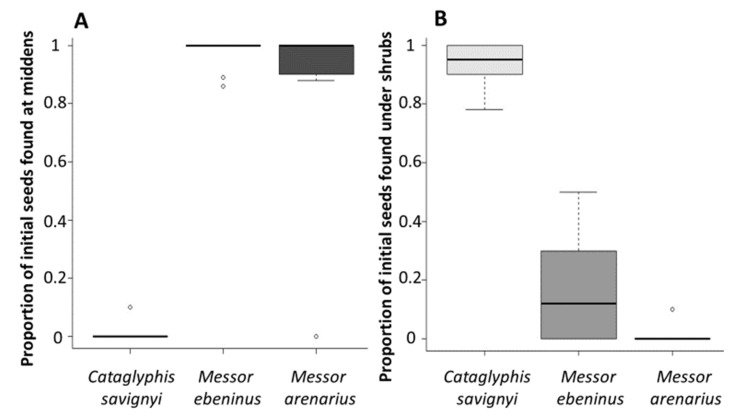
Habitats of final seed location following redispersal by each ant species: (**A**) Location relative to middens. (**B**) Location relative to shrubs. Each boxplot represents the median (black horizontal line), 25, and 75 quartiles. The error lines as described in Figure 1.

**Figure A1:**
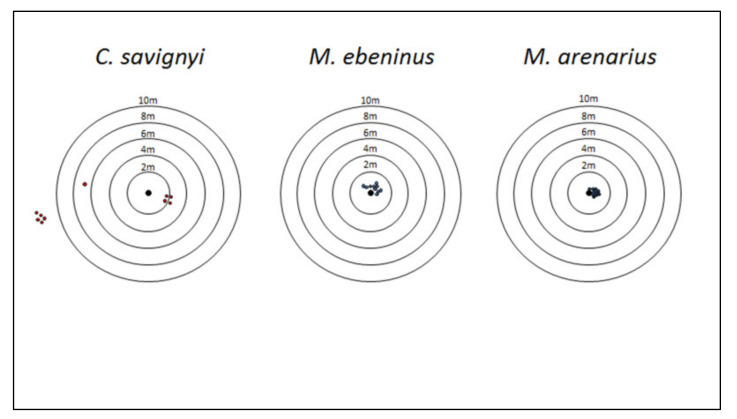
A graphic presentation of actual redispersal spatial patterns around a typical nest of each of the three ant species studied. The north is at the top of the diagram. The nest opening is the black point at the center of each diagram, and the circles contouring from it outwards are two meters apart one from the other. Each blue (for granivores) or red (for scavengers) point is the location of a seed removed from the nest.

The ant species differed in their redispersal behavior, as well as in the characteristics of the final locations of the seeds. The scavenger, *C. savignyi*, redispersed the seeds more frequently and much farther than both granivore species ($\chi_{\left(2\right)}^{2}\;=\;35.64,\;p<0.001$, Figure 2, Table A2 and Table A3). Granivore species rarely redispersed the seeds, discarding most of them at the middens (*M. ebeninus*–97.44%, *M. arenarius*–96.20%), significantly more so than scavengers ($\chi_{\left(2\right)}^{2}\;=\;51.81,\;p<0.001$, Figure 3A, Table A2 and Table A3). Furthermore, the seeds redispersed by the scavengers were aggregated. Out of the 100 seeds that were presented to *Catglyphis* colonies, 94 seeds were redispersed (Figure 3B). Six of the 94 seeds that were redispersed were located in open sites, while the rest (88/94 seeds) were deposited under shrubs. Typically, seeds redispersed from a single *Catglyphis* colony ended up in two or three aggregates (an example is given in Figure A1).

### 3.2. Affinity of Sternbergia Clusiana to Shrubs

Overall, we studied 173 plots, each the size of 1m × 1m in three desert sites. All the plots had *S. clusiana* mature plants in them. In 91 of these plots, there were fruit-bearing plants, and in 43 of the plots, seedlings were present. The distribution of the individuals within plots with respect to the presence of shrubs indicated that *S. clusiana* were more likely to be found under shrubs than expected by random, given the proportion of shrub cover. This was true for all three life stages in two sites (Boker and Lehavim), and true for mature and fruiting plants in the third site (Yeruham), (Table 1). The number of plots with seedlings in that third site was extremely small–seven plots only, thus preventing any meaningful statistical testing for that life stage in that site.

## 4. Discussion

The most well-documented ant–seed interaction in deserts is between harvester ants and seeds, which often results in seed consumption, but under specific conditions, it may result in beneficial seed dispersal [14,21,49]. However, it is largely unknown whether ant guilds other than harvester ants interact with seeds in desert systems, and even less so whether they have any effect on seed fate. In a previous study, we found that when given the opportunity, scavenger ants in desert sites are much more likely to remove the seeds of *S. clusiana* (first phase of myrmecochory) than co-occurring granivorous ants [34]. In the current study, we found that there also are significant differences between the guilds in the post seed-removal phases. Combined, these studies provide a comprehensive example of the range of dispersal services that different ant guilds deliver to a myrmecochorous plant in a desert ecosystem. Furthermore, our results highlight the importance of post-removal dispersal stages in determining seed fate. As far as we know, this is the first time that all stages of the dispersal process of a myrmecochorous seed-dispersal interaction are documented within the same system.

The scavenger species, *C. savignyi*, clearly exhibits characteristics that would make it a more effective disperser of *S. clusiana* seeds than the granivorous species. In addition to the higher seed removal rates that we observed previously [34], *C. savignyi* also exhibited seed handling behaviors that are beneficial for the plant. It was the only ant species in our study that exhibited both low seed predation and removed the elaiosome from high proportion of seeds. The granivorous species exhibited either high seed predation (*M. arenarius*) or failed to remove the elaiosome (*M. ebeninus*). That low seed predation is advantageous for the plant is obvious. Elaiosome removal also has a positive effect on plant fitness. It reduces further seed predation [25,26,50] and promotes germination success by removing inhibitors like bacteria or fungi [51,52] (but see [27,53]).

The ant species also differ with respect to redispersal. The granivore species in our study did not redisperse seeds, but rather discarded the seeds at the middens surrounding the nest opening, where seeds of other plant species accumulate as well. The middens of granivorous ants in general [2,3,5,21,54], and of the genus *Messor* in particular [55,56,57], are regarded as nutrient-enriched sites, which may have a positive effect on plants. However, despite this enrichment effect, the accumulation of many seeds in those middens also may lead to intense inter- and intra-specific competition that eventually sums to a negative effect on plant fitness [58]. The consequences of such directed dispersal of *S. clusiana* seeds to middens of *Messor* sp. was not studied directly. Nevertheless, of the several thousand individuals of *S. clusiana* that we observed, only two were recruited to middens of the *Messor* species. In contrast to the granivores, the scavenging species *C. savingyi* exhibited clear redispersal behavior. Not only did it redisperse most of the seeds that were initially taken to the nest, but this redispersal was disproportionally directed towards sites under shrubs, which were found to support high densities of the plant population at various life stages.

Redispersal of myrmecochorous seeds has seldom been documented, but when it has, the focus has been on scavenging ant species that serve as keystone dispersers in their respective ecosystems. Examples include *Aphaenogaster rudis* in eastern North America [29], *Rhytidoponera metallica* and *Iridomyrmex viridiaeneus* in Australia [59,60], and *Myrmica ruginodis* in Europe [61]. Furthermore, to the best of our knowledge, non-random redispersal has rarely been reported [23], while directed redispersal of a myrmecochorous seed has never been reported. The fact that those ant species that were documented redispersing myrmecochorous seeds also exhibit other traits and behaviors that render them efficient seed dispersers, suggests that redispersal behavior is a feature that is part of a keystone disperser syndrome.

The main benefits attributed to redispersal are the further displacement of seeds from the parent plant (distance dispersal) and the reduction of inter- and intra-specific competition that is experienced on the nest middens. Such a reduction in seed aggregation is mainly effective if redispersed seeds are scattered to various locations, either randomly [29,62] or even non-randomly, as long as the resulting densities are lower than those prior to redispersal [23]. The non-random redispersal of *S. clusiana* seeds to shrub patches that we observed in our study often reduces intraspecific competition, as seeds from each *C. savignyi* colony were redispersed to 1–5 shrubs located at least a few meters from each other (Figure A1). In addition, the environmental conditions experienced by seeds redispersed to the shrub patches are different and possibly better than those at the nest mound. Theoretically, the placement of several seeds in a shrub patch may lead to a modest seed aggregation (and potentially higher competition) compared to a random scattering of seeds [58,63]. Nevertheless, the observations of the positive affinity between shrubs and the various life stages of *S. clusiana* suggest a relatively weak long-term effect of intraspecific competition at the shrub patches, if at all.

Shrubs in arid regions serve as ecosystem engineers and landscape modulators [64,65], and function as fertile islands that enhance establishment and growth of many herbaceous species, due to nutrient enrichment, improved water regime, and seed trapping [35,36,66]. Due to the difficulties in long-term monitoring of *S. clusiana,* the role of shrubs in our study system could only be addressed indirectly. Specifically, we showed that overall, there was a high affinity between individuals of *S. clusiana* at different life stages (seedlings, mature and fruiting plants) and shrubs. This non-random distribution can be the result of the initial disproportional (re)dispersal of seeds to shrub habitats or indicate that the shrubs can facilitate germination and establishment of *S. clusiana* in this harsh desert environment. Some support for the second explanation comes from observations that, in sites where scavenging ants are locally absent (and consequently redispersal is most likely absent as well), individuals of *S. clusiana* are still distributed mainly under shrubs [34]. However, in order to test directly whether the non-random distribution of *S. clusiana* is only a legacy of non-random dispersal to shrubs or indicates higher microhabitat quality, future research should include either experiments or long-term demographic study comparing germination and establishment under shrub canopy to those in open habitat.

While there are a few plausible explanations for the benefits that a plant may gain from being dispersed under shrubs, the incentives for the scavenging ants to carry seeds disproportionally to this microhabitat are not clear. Removing any waste materials, including seeds, away from the nest entrance could be part of a strict sanitation behavior that is typical of scavenging ants [17,29,60,67]. Waste-disposal under shrubs probably increases its decomposition rates in desert areas [35], thus reducing both sanitation risks and the attractiveness of the waste, to competitors and predators of the ants. It is also possible that the *C. savignyi*, which often use objects like shrubs for their navigation, simply deposit the seeds in the first clear landmark, which happens to be a nearby shrub. Another intriguing possibility relates to the preferred nesting sites by scavenging ants in relation to those of other ants. Large scavenging ants, such as *Cataglyphis spp.*, locate their nests in open sites, which reduces competition with more dominant ant species [68,69]. In our study site, *M. ebeninus* and some dominant omnivorous ant species (*Tapinoma* and *Monomorium spp.*, personal observation), exhibits a significant preference to build nests under shrubs [43].

## 5. Conclusions

We showed clear differences in the post-removal behaviors by two guilds of desert dwelling ants and how these differences govern seed fate and recruitment opportunities of a myrmecochorous plant. Specifically, granivorous ants treat myrmecochorous seeds as they treat other seeds, consuming most and rejecting unconsumed or partially consumed seeds in the midden. Scavengers, on the other hand, did not harm the seeds during collection, and redispersed them in a non-random manner to sites that offer potential benefits to plant future success. Our study sheds light on the major role of ant guilds in seed dispersal in desert ecosystems, while stressing the importance of following the post-removal stages of seed dispersal to determine actual fate of the seed.

## Figures and Tables

**Table 1:** Results of the paired-sampled t-tests for each life stage of *S. clusiana* in all three sites. For each plot containing a specific life stage, the t-test compares the number of individuals of the specific life stage occurring under shrub with the expected number of individuals at that life stage, assuming these are distributed in proportion to the shrub cover in that plot. In addition, means and standard errors of the difference between expected and observed are indicated.

Site	Seedlings	Mature Plants	Fruiting Plants
Boker	t_(10)_ = 4.74, *p* = 0.001	t_(34)_ = 3.93, *p* < 0.001	t_(23)_ = 4.96, *p* < 0.001
	$\bar{x}$ = 1.21, SE = 0.26	$\bar{x}$ = 6.75, SE = 1.72	$\bar{x}$ = 1.00, SE = 0.20
Yeruham	t_(7)_ = 1.14, *p* = 0.299	t_(48)_ = 4.37, *p* < 0.001	t_(13)_ = 3.00, *p* = 0.01
	$\bar{x}$ = 0.28, SE = 0.25	$\bar{x}$ = 4.47, SE = 1.02	$\bar{x}$ = 0.40, SE = 0.13
Lehavim	t_(24)_ = 4.15, *p* < 0.001	t_(88)_ = 6.09, *p* < 0.001	t_(52)_ = 6.67, *p* < 0.001
	$\bar{x}$ = 1.02, SE = 0.25	$\bar{x}$ = 3.11, SE = 0.51	$\bar{x}$ = 0.78, SE = 0.12

## Data Availability

Data are available at Dryad: Giladi, Itamar (2021), Seed re-dispersal experiments, Dryad, Dataset, https://doi.org/10.5061/dryad.3xsj3txfx.

## Appendix A

**Table A1:** Study sites where the distribution of *S. clusiana* with respect to shrubs was documented. Climate data retrieved from the Israel Meteorological Service website (1). Lithology data was retrieved from the geological map of Israel (2).

Site	Region	Location (Long. Lat.)	Altitude (Meters A.S.L)	Annual Rainfall (mm, 1981–2010)	Average Temp. (°C, January/July, 1995–2009)	Lithology	Aspect and Slope
Lehavim	Northern Negev	34°49′42″E 31°22′04″N	350	297	12.1–27.8	Eocene chalk + Quaternary Loess	Various aspects and slopes
Yeruham	Central Negev	34°53′16″E 30°58′27″N	530	98	9.7–26.2	Cenomanian limestone	North-facing rocky outcrops
Boker Ridge	Central Negev	34°46′15″E 30°54′59″N	490	93	9.9–26.2	Turonian limestone	North-facing rocky outcrops

**Table A2:** Summary of the GLMs testing the effect of ant species on variables of seed fate and final location—presented are the χ^2^ values with df in parentheses; significant effects are in bold.

Dependent Variable	Seed Predation Rate	Elaiosome Removal Rate	Redispersal Distance	Rate of Discarding in Middens	Rate of Redispersal under Shrubs
Intercept	**50.70**_(1)_	**38.13**_(1)_	0.19_(1)_	2.92_(1)_	**7.97**_(1)_
Ant species	**25.07**_(2)_	**85.74**_(2)_	**35.64**_(2)_	**51.81**_(2)_	**83.32**_(2)_

**Table A3:** Summary of the GLMs testing the effect of each ant species on variables of seed fate and final location—coefficient values (marked by Bs) are calculated relative to *Messor arenarius* (which is included in the intercept); significant effects are in bold. All the χ**^2^** tests that appear in the table has a df = 1.

Dependent Variable	Seed Predation Rate		Elaiosome Removal Rate		Redispersal Distance (Log-Transformed)		Rate of Discarding in Middens		Rate of Redispersal under Shrubs	
	B (SE)	χ^2^ (DF)	B (SE)	χ^2^ (DF)	B (SE)	χ^2^ (DF)	B (SE)	χ^2^ (DF)	B (SE)	χ^2^ (DF)
Intercept	0.77 (0.24)	**10.14**	2.33 (0.40)	**34.66**	−1.06 (0.88)	1.43	3.23 (0.59)	**30.15**	14.36	**18.74**
*Cataglyphis savignyi*	3.76 (1.03)	**13.24**	0.78 (0.65)	1.47	3.07 (0.88)	**12.08**	−7.77 (1.17)	**44.42**	7.04	**41.65**
*Messor ebeninus*	3.57 (1.04)	**11.92**	3.46 (0.48)	**53.01**	0.54 (1.02)	0.27	0.41 (0.93)	0.19	2.84	**7.32**
